# Can Extra Daytime Light Exposure Improve Well-Being and Sleep? A Pilot Study of Patients With Glaucoma

**DOI:** 10.3389/fneur.2020.584479

**Published:** 2021-01-15

**Authors:** Aki Kawasaki, Morgane Udry, Mohamad El Wardani, Mirjam Münch

**Affiliations:** ^1^Hôpital Ophtalmique Jules Gonin, Fondation Asile des Aveugles, Department of Biology and Medicine, University of Lausanne, Lausanne, Switzerland; ^2^Ophthalmology Department, Calderdale and Huddersfield NHS Foundation, Huddersfield, United Kingdom; ^3^Sleep/Wake Research Centre, Massey University, Wellington, New Zealand

**Keywords:** light therapy, pupil, glaucoma, retinal ganglion cells, melanopsin, sleep, mood, circadian

## Abstract

Glaucoma damages retinal ganglion cells, including intrinsically photosensitive retinal ganglion cells (ipRGCs). These cells modulate various non-visual physiological and psychological functions which are modulated by light. In patients with glaucoma, we assessed the effect of daily bright light exposure (LE) on several melanopsin-dependent functions, such as the pupil constriction, circadian rest-activity cycles, sleep and subjective well-being including relaxation, alertness and mood. Twenty patients participated in the study (9 women, 11 men, mean age = 67.6 ± 7.5 y). Pupillometry was performed before the LE weeks and repeated on the last day of LE. The post-illumination pupil response (PIPR) was calculated as a proxy for melanopsin-dependent activation. Participants continuously wore an activity monitor and self-assessed sleep quality, well-being and visual comfort for 7 days before and during 4 weeks of daily bright LE (30 min to 10,000 lux polychromatic bright white light). After the LE, there was a significantly greater PIPR and higher subjective sleep quality when compared to the pre-LE week (*p* < 0.05), but no significant changes in 24-h rhythms or sleep parameters. A greater PIPR was correlated with an increase in circadian amplitude and higher inter-daily stability (derived from rest-activity cycles; *p* < 0.05). In a small group of patients with glaucoma, scheduled daily bright light exposure could improve subjective sleep quality. These findings highlight the importance to evaluate and maintain non-visual functions at different levels in patients with progressive loss of ipRGCs.

## Introduction

Glaucoma is a common optic neuropathy that results in retinal ganglion cell loss. In patients with advanced glaucoma, loss of intrinsically photosensitive retinal ganglion cells (ipRGCs) has been demonstrated (1). The ipRGCs express melanopsin and synapse centrally to modulate a variety of non-visual physiological functions such as the pupil, circadian rhythms, alertness and sleep (2). Reduced capacity to entrain to light and was first shown in glaucomatous rats (3, 4). Extending those findings to patients with glaucoma, even those with mild visual dysfunction may demonstrate abnormal light responses including reduced acute alerting effects to light, reduced light-induced suppression of nocturnal melatonin and reduced pupil light reflex to selected light stimuli as these are effects mediated by melanopsin (5–10). Glaucoma patients have also been shown to have a higher prevalence for impaired executive daytime functions, depressive symptoms, impaired mood (11, 12) and anxiety (13) and clinical implications have been discussed [for a reviews see (14–16)].

Additionally, there is evidence that patients with glaucoma have greater daytime sleepiness and/or decreased sleep quality compared to age-matched control subjects (17–19). A large cross-sectional study with more than 6,700 patients reported an association between glaucoma and very long sleep duration (20). Lanzani et al. reported increased wakefulness with lower sleep efficiency at night (21) and similarly Gubin et al. found later bed times and shorter sleep duration in primary open angle glaucoma patients (22) suggesting a higher prevalence for sleep disturbances in patients with glaucoma (13, 23, 24). There is some evidence that obstructive sleep apnea might be a systemic risk factor for glaucoma (25–28), even though not all study reports confirmed this (20, 29).

One factor contributing to the mechanism of impaired sleep in glaucoma may be related to loss of ipRGCs and consequently, a reduction of the melanopsin-mediated light signaling to the suprachiasmatic nuclei (SCN), the principal biological pacemaker in the hypothalamus. Such reduced light input due to glaucoma-related loss of ipRGCs (1) may be further amplified by physiologically occurring age-related ipRGC loss (30), and physiologic changes of circadian rhythm regulation and sleep (31, 32), as glaucoma generally affects elderly persons (30, 33). Since light is the principal external zeitgeber on which the biological circadian clock aligns to the 24-h day–night cycle, the combination of inadequate light exposure during daytime and loss of ipRGCs together may reduce the effectiveness of light as a zeitgeber in patients with glaucoma. The potential consequence is impaired circadian entrainment and adverse influence on well-being, mood, 24-h rest-activity cycles and sleep (34).

We hypothesize that it is possible to enhance the external zeitgeber strength by increasing daytime light exposure in patients with glaucoma. This, in turn, will have beneficial effects, particularly on sleep, 24-h rest-activity cycles, mood and well-being. Because we presume the effect of light is mediated through the melanopsin system, we also hypothesize that melanopsin activity will change and will be detectable in the pupil response (6, 35, 36). Thus, the aim of this study is to assess how scheduled additional bright light exposure during daytime might impact pupil light reflex, sleep, circadian rest-activity cycles and subjective mood and well-being in patients with glaucoma.

## Methods

### Participants

Study participants were recruited from patients actively treated for glaucoma at the Hôpital Ophtalmique Jules-Gonin in Lausanne, Switzerland. Patients with the following conditions were excluded: unilateral glaucoma, congenital glaucoma, non-glaucomatous optic neuropathy, diabetes, past or present cancer, sleep apnea, diagnosed mood disorder, recent use of recreational drugs (e.g. cannabis, cocaine), alcohol dependency or pregnancy. Other exclusionary criteria included night shift work within the last three months, current use of sleeping pills or travel across a time zone <1 month before study participation. All participants provided oral and written informed consent and were approved by their treating ophthalmologist to participate in the study. The study was conducted at the Hôpital Ophtalmique Jules Gonin, Lausanne, Switzerland according to the tenets of the Declaration of Helsinki and received authorization from the local ethical review board for human research (Commission d'Ethique de Recherche sur l'être humain de Canton de Vaud, Switzerland no. 2018-01749). All participants were clinically examined at least once by their treating ophthalmologist during or after study participation.

Twenty patients with glaucoma participated in the study. They were 9 women and 11 men, mean age = 67.55 ± 7.45 years, age range 53 to 79 years. The types of glaucoma were primary open angle (*n* = 10), primary angle closure (*n* = 8) and pseudo exfoliative glaucoma (*n* = 2). Eight patients had undergone laser iridoplasty, and seven patients had undergone glaucoma filtration surgery. Ten patients had undergone uncomplicated cataract surgery with intraocular lens replacement. All procedures had been performed at least 6 months prior to study participation. These procedures did not affect the iris sphincter muscle contractibility, as assessed at the slit lamp.

### Study Design

From the medical charts, the following ophthalmologic information was extracted for each eye: best-corrected visual acuity, mean defect of the automated visual field, and thickness of the peripapillary retinal nerve fiber layer (RNFL) from the optical coherence tomography (see Supplementary Table 1). If the ophthalmologic examination dated more than 6 months before study participation, the participant underwent these tests at the first study session. The study comprised 4 sessions at the Hôpital Ophtalmique Jules Gonin (46.5197° N, 6.6323° E), Switzerland over 5 consecutive weeks and was conducted from January to July 2019.

At session 1, the participant came to the hospital, gave informed written consent and completed six standardized questionnaires: Horne-Ostberg [HO; (37)], Munich Chronotype Questionnaire [MCTQ; (38)], Epworth Sleepiness Scale [ESS; (39)], Pittsburgh Sleep Quality Index [PSQI; (40)], Seasonal Pattern Assessment Questionnaire [SPAQ; (41)] and the Beck Depression Inventory [BDI; (42)]. These questionnaires were used to assess chronotype, daytime sleepiness, habitual sleep quality (during the preceding 4 weeks), seasonal effects and depressive symptoms (see Supplementary Table 2). At the end of session 1, the participant received an activity monitor (Actiwatch L, Camntech Cambridge, UK) to be continuously worn on the non-dominant wrist during the next 5 weeks in order to record 24-h rest-activity cycles. The participant was instructed to maintain a daily sleep log (bedtime, wake up time) and note subjective sleep quality (1 = very bad sleep, 10 = excellent sleep) every morning. Also, the participant made a daily self-assessment of well-being and visual comfort using a visual analog scale (see section Methods).

After 1 week, the participant came again to the hospital (session 2) and underwent pupillometry (see below). At the end of session 2, the participant was given a commercially available table-based light box (EnergyUp / HF3419™ Philips, The Netherlands) for home use. The light source emits a diffuse polychromatic bright white light at 10,000 lux [see light measures according to the CIE Standard, (CIE S 026) in the Supplementary Table 3]. The participant was instructed to sit face-forward at 50 cm distance from the light box every morning around the same time for 30 min over the next 4 weeks. No additional change of their usual lifestyle was requested.

After 2 weeks, participants were asked to come to the laboratory for session 3 which served to verify compliance with the light exposure at home and to download rest-activity data of the first 3 weeks. The activity monitor was then returned to participants. After another 2 weeks, participants came to the laboratory for the final session 4 to complete one questionnaire (second PSQI) and to undergo the second pupillometry. Participants also returned the completed subjective assessments, the activity monitor and the light box. An overview of the study design is shown in Supplementary Figure 1.

### Pupillometry

In this study, pupillometry was used as a quantitative method to assess the functionality of the melanopsin-signaling pathway. The pupillometer used for this study was a monocular device (Neurolight, IDMed, Marseille, France) which presented light stimuli and recorded the pupil of the stimulated eye at a sampling frequency of 67 Hz. The light stimulus was a narrow bandwidth short-wavelength light stimulus (peak wavelength at 470 nm; “blue”) having a 1s duration. Two brightness intensities (luminance: 56 cd/m^2^ and 170 cd/m^2^) were used with intention of activating rod and cone photoreceptors as well as the melanopsin photopigment of ipRGCs, as described in previous studies (36, 43). The pupil test started with 3 s of darkness followed by a blue light stimulus at lower luminance (56 cd/m^2^) and then a second blue light stimulus at higher luminance (170 cd/m^2^). The inter-stimulus dark interval was 15 s. By convention, the right eye was always tested first. Both eyes were tested under photopic condition (prior adaptation to room light at 150 lux at a vertical direction at eye level for 10 min), and scotopic condition (adaptation to darkness 0 lux for 20 min). The non-tested eye was covered by the participant's hand. Pupils were measured twice, once at session 2, that is, before the start of scheduled daily bright light exposure from the light box and denoted as pre-light exposure (= pre-LE), and again at session 4, that is, after 4 weeks of daily bright light, denoted as post-light exposure (= post-LE).

From the pupillometer device, raw tracings were downloaded with NL Viewer Software (v 1.2, IDMED, Marseille, France). All recorded tracings were visually inspected and artifacts from movement or blinking were removed by linear interpolation in Excel (Microsoft Office, v 7). The following outcome parameters were determined for each pupil recording: baseline pupil size (BL), maximum contraction amplitude (MCA), post-illumination pupil response (PIPR). The BL was calculated as the averaged pupil size during 0.25 s before the first light stimulus. Thereafter, pupil size was normalized by expressing absolute pupil size in mm as a percentage of BL in mm (%). Normalization of pupil size was important because BL of the pupil before the second stimulus was often slightly smaller than BL before the first stimulus. The MCA was identified as the smallest pupil size within 3 s from light stimulus onset and expressed in % as the difference from the baseline pupil size [maximum contraction amplitude = (1 – smallest relative pupil size) ×100%]. The PIPR (in %) was amount of pupil constriction 6 s after the stimulus light was terminated and calculated as: [(1 – relative pupil size at 6 s after light offset) ×100%].

### Rest-Activity Recordings for 24-h Rhythms and Sleep

After downloading the rest-activity data (sampling frequency = 1 min), all recordings across 5 weeks were joined to one file per participant and each 24-h epoch was visually inspected. Any 24-h days with more than 3 h of missing data were excluded from further analysis (44). Missing data of <3 h were edited with the mean activity of 24-h using the software Sleep Analysis (v7, Camntech, Cambridge, UK) with an inbuilt algorithm to detect sleep (at medium sensitivity of the device). Using the non-parametric circadian rhythm analysis [NPCRA; (45)] implemented in the software (Sleep Analysis, v7), the following parameters were assessed: intra-daily variability (IV), inter-daily stability (IS), the absolute amplitudes derived from rest-activity oscillations (in arbitrary units) of 24 h (absolute amplitude, AMP) and of the 5 h with least activity (L5) and the 10 h of highest activity (M10) during a 24 h period. The M10 onset typically occurs during daytime and L5 onset typically occurs during nighttime. We also assessed the onset clock time of the 10 consecutive hours of greatest activity (M10on), the onset clock time of 5 consecutive hours of least activity (L5on). The IV evaluates the frequency of transitions between rest and activity per day, which is an indicator of fragmentation of the 24-h rest-activity rhythm. A lower IV score reflects less rest-activity rhythm fragmentation. The IS evaluates the strength of coupling between the rest-activity rhythm (45). A higher IS score is considered as greater invariability between days. The relative amplitude (RA) derives from the difference between M10 minus L5 expressed relative to the 24 h activity, where a higher RA indicates more consolidated high daytime and low nighttime activity.

In addition, bedtime (clock time), get-up time (clock time) were determined semi-manually from the activity recordings (and sleep logs). The sleep analysis tool of the same software (Sleep Analysis, v7) was used to determine the following sleep parameters, derived from rest-activity recordings (sampling frequency = 1 min epochs): time in bed (hours), sleep duration (hours), actual wake time during scheduled sleep (hours), sleep efficiency (sleep duration / time in bed x 100; %), sleep latency (time range between bed time until the first episode of consolidated sleep, as assessed by the sleep software).

Subjective sleep quality was daily assessed for 5 weeks after get-up (on the sleep log) by indicating a number between 1 (very bad sleep) and 10 (excellent sleep) on a paper-based version and individual scores were averaged per week.

### Visual Analog Scales for Subjective Well-Being and Visual Comfort

Participants were instructed to complete a paper-based visual analog scale (VAS) for subjective well-being and visual comfort every day around the same clock time in the morning during 5 weeks. For subjective well-being, the VAS consists of a vertical line from 0 and 100 mm and represents the extremes of relaxation, physical comfort, alertness: 0 mm = extremely relaxed/100 mm = extremely tense; 0 mm = physically comfortable/100 mm = physically not at all comfortable; 0 mm = extremely alert/100 mm = extremely sleepy; 0 mm = bad mood/100 mm = very good mood. The participant indicated the current state of these parameters by marking a vertical line on the scale.

For visual comfort, five specific items of lighting were assessed [adapted from the Office Lighting scale (46)] and presented on a VAS between 0 mm and 100 mm: (1) I like the lighting (0 mm)/I do not like the lighting in this room (100 mm); (2) the lighting is pleasant (0 mm)/the lighting is not pleasant at all (100 mm); (3) this room is too bright (0 mm)/this room is too dark (100 mm); (4) there is too much light to read/work properly (0 mm)/there is not enough light to read/work properly (100 mm); and (5) the glare in this room is imperceptible (0 mm)/the glare in this room is intolerable (100 mm). The participant indicated the current state of these items by marking a vertical line on the VAS scale. For analysis, a weekly average was determined from the paper-based daily assessed VAS, for each parameter of well-being (relaxation, physical comfort, alertness, mood) and for five items of visual comfort.

### Statistics

For differences between ophthalmological screening measures (left and right eyes) two-tailed *t*-tests were applied. For pupil data, a mixed linear model with the factor WEEK was applied to compare the recording of the pre-LE with the recording after the 4 weeks (= post-LE) with daily scheduled light exposure with AGE, SEX and their interactions also added to the model. For all analyses, AGE was used on dichotomized variables based on median split (median = 69.5 years). For continuous rest-activity and sleep data as well as subjective assessments, all averaged per week, a generalized linear regression model was used with the repeated factor WEEK (i.e., averages of pre-LE week 1 and each of the LE weeks 2-5) and the fixed factors AGE, SEX, and their interactions. *Post-hoc* tests were performed with the Tukey-Kramer, adjusted for multiple comparisons. For tables, averaged values of the pre-LE and the average of LE-weeks 2–5 are shown. For correlations Pearson's correlation was used. The SAS software (v. 9.4.; SAS Institute Inc., Cary, NC, USA) was used for statistical analyses.

## Results

### Participants

There was no significant interocular difference for visual acuity (VA) and for mean deviation (MD) on automated perimetry (2-sided *t*-tests; *p* > 0.09). The VA ranged from 0.3 to 1.5, mean 0.85 ± 0.17 for both eyes (Supplementary Table 1). The mean MD for both eyes was 5.41 dB ± 3.17 for both eyes, range −0.9 to 20 dB. On OCT, the peripapillary retinal nerve fiber layer (RNFL) thickness was abnormal or borderline in all but one eye of 19 patients as OCT data from one patient was not available. The descriptive statistics for the questionnaires (MCTQ, ESS, HO, SPAQ, BDI and PSQI) are shown on Supplementary Table 2. The post-LE PSQI was not significantly different (*p* = 0.67; generalized linear model) from pre-LE.

### Pupillometry

There were small, but statistically significant differences between left and right eyes in scotopic baseline pupil size (Supplementary Table 4). Because the right eye was always tested first and reflects the most accurate photopic and scotopic adapted condition, we used the pupil results from right eyes for further analyses. Older participants had a smaller baseline pupil size before the first light stimulus under scotopic conditions (main effect of AGE; *F*_1, 20_ = 5.49; *p* = 0.03; mean ± SD older: 4.83 mm ± 0.55 mm; younger participants; 5.38 mm ± 0.84 mm). As expected, the pupil response (MCA and PIPR) to the 1s light stimulus at higher luminance (170 cd/ m^2^) was greater (and BL pupil size smaller) than the response to the 1s light stimulus at lower luminance (56 cd/ m^2^; Table 1; *F*_1, 20_ > 4.9; *p* < 0.038) under photopic and scotopic testing conditions.

**Table 1:** Mean values (± SD) across patients (for right eyes) for baseline pupil size (BL), maximum contraction amplitude (MCA) and post-illumination pupil response (PIPR).

	**Pre-LE week**	**Post-LE weeks**
BL photopic low (mm)	3.50 (0.73)	3.59 (0.72)
BL photopic high (mm) ∧	3.41 (0.73)	3.51 (0.72)
BL scotopic low (mm)	5.07 (0.76)	5.14 (0.77)
BL scotopic high (mm) ∧	4.61 (0.73)	4.64 (0.64)
MCA photopic low (%)	23.86 (8.79)	23.88 (7.97)
MCA photopic high (%) ∧	29.33 (8.56)	29.29 (7.98)
MCA scotopic low (%)	40.74 (6.11)	40.32 (6.37)
MCA scotopic high (%) ∧	46.02 (6.51)	46.40 (6.39)
PIPR photopic low (%)	4.17 (3.33)	3.26 (2.87)
PIPR photopic high (%) ∧	9.48 (5.90)	9.07 (3.92)
PIPR scotopic low (%)	19.14 (8.66)	20.35 (10.15)
PIPR scotopic high (%) ∧, ^*^	25.07 (9.53)	28.71 (10.46)

*BL in mm = 100 %; MCA in % difference to BL and PIPR in % difference to BL. All mean values are shown for recordings before the pre-light exposure (pre-LE) week and the post-light exposure (post-LE), that is, the pupil recording immediately at the end of 4 weeks of scheduled daily bright light exposure. Photopic low = 1s short-wavelength narrow bandwidth light stimulus (= blue light stimulus) at lower luminance (56 cd/m^2^) under photopic conditions (i.e., after 10 min of room light adaptation); photopic high = 1s blue light stimulus at higher luminance (170 cd/m^2^) under photopic conditions; scotopic low = 1s blue light stimulus at lower luminance (56 cd/m^2^) under scotopic conditions (i.e., after 20 min of dark adaptation); scotopic high = 1s blue light stimulus at higher luminance (170 cd/m^2^) under scotopic conditions; ∧ = p < 0.05, indicate differences between light stimuli used for pupil measures at higher (170 cd/m^2^) and lower luminance (56 cd/m^2^)*.

* *= p < 0.05, shows difference between pupil tests pre-light exposure and post-light exposure; n = 20*.

For all pupil parameters related to the 1s light stimulus at lower luminance (56 cd/ m^2^) there was no statistically significant difference between the pre-LE and post-LE (i.e., after 4 weeks of daily scheduled bright light exposure; Table 1; *F*_1, 20_ <1.5; *p* > 0.24).

**Figure 1:**
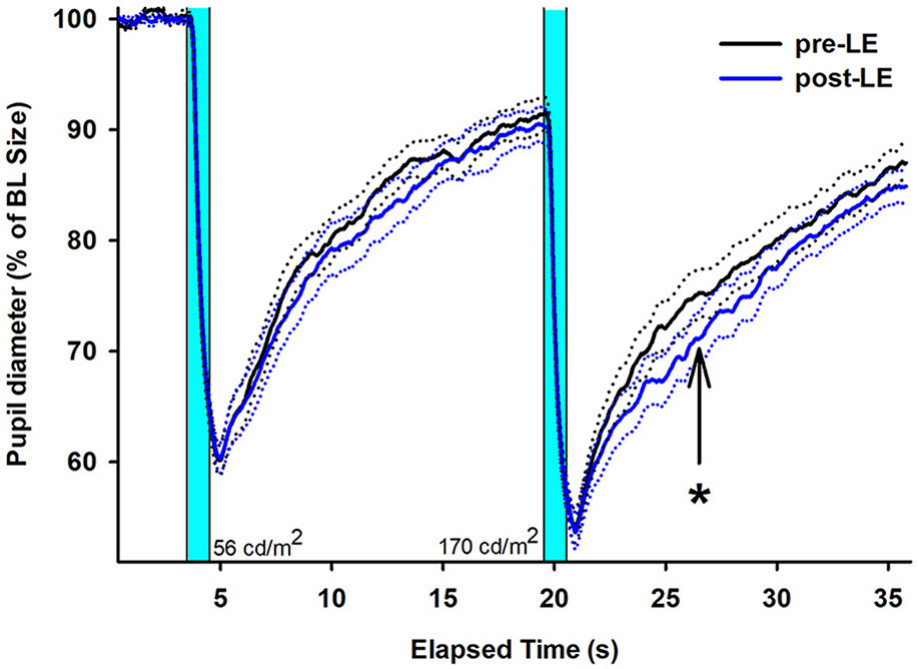
Mean (solid lines) pupil recordings for 20 subjects with glaucoma (right eyes, SEM shown as dotted lines). The pre-LE recordings (taken in week 1) are shown in black; the post-LE recordings (taken immediately after 4 weeks after daily scheduled bright light exposure) are shown in blue (SEM = dotted black and blue lines). LE = light exposure. The two vertical bars indicate when the two blue light stimuli are given (1s duration). The first 1s light stimulus is at lower (56 cd/m^2^) and the second 1s light stimulus at higher luminance (170 cd/m^2^). The arrow and asterisk designate the PIPR after the second (brighter) light stimulus with a significant difference between pre- and post-LE measures (*p* < 0.05). The data is shown relative to baseline (= 100 %).

As a main finding of this study, the scotopic post-LE PIPR to the 1s light stimulus at higher luminance (170 cd/ m^2^) was, however, significantly greater compared to pre-LE PIPR (Table 1 and Figure 1). The increase in the scotopic post-light PIPR was, on average, 13% and was observed in 14 out of 20 patients (main effect of WEEK; *F*_1, 20_ = 6.02, *p* = 0.02, Supplementary Figure 2).

### 24-h Rest-Activity Cycles and Sleep

Results from rest-activity recordings were averaged per week (see also methods; for one participant, the first 2 weeks of rest-activity data with daily bright light exposure were missing, and for one participant the rest-activity data during the last LE week was only available for 2 days). For 24-h rest-activity variables, there was no significant difference between the pre-LE week and the LE weeks values (Table 2; *F*_4, 60_ <1.8, *p* > 0.17), except for the RA which dropped in week 5 (0.84 ± 0.09) when compared to week 4 (0.87 ± 0.06; main effect of WEEK; *F*_4, 59_ = 3.1, *p* = 0.02). However, there was no difference for RA between the pre-LE week and any single week during the daily light exposure (*p* > 0.48).

**Table 2:** Mean values (and SD in brackets) for variables from the circadian rest-activity cycles.

**Variable**	**Pre-LE week**	**LE weeks**
IS	0.58 (0.11)	0.59 (0.11)
IV	0.82 (0.28)	0.82 (0.22)
L5	1,434 (759)	1,600 (926)
L5on	24.78 (1.30)	24.92 (1.33)
M10	21,477 (8,100)	21,979 (8,450)
M10on	8.83 (1.63)	8.67 (1.71)
AMP	20,043 (7,935)	20,378 (8,271)
RA ∧	0.87 (0.07)	0.86 (0.08)

*IS = inter-daily stability; IV = intra-daily variability; L5 = 5 h with lowest activity; L5 onset = onset time of the 5 h with least activity; M10 = 10 h with highest activity oscillations; M10 onset = onset hours of the 10 h with greatest activity. AMP = absolute amplitude; RA = relative amplitude (see method section for more explanations). Averaged values across participants are shown with SD (in brackets) for the pre – LE week and the LE weeks (i.e., the 4 weeks with scheduled daily bright light; weeks 2–5). ∧ = p < 0.05, indicating higher RA during week 4 than 5 (main effect of week; for mean values in weeks 4 and 5 separately see also text); n = 20*.

The sleep variables did not reveal significant changes between pre-LE and LE weeks for any of the parameters (see means ± SD for the pre-LE week and averaged LE weeks on Table 3). There was a decrease in sleep efficiency from week 3 and 4 to week 5 (main effect of WEEK; *F*_4, 62_ = 3.3, *p* = 0.02) from 87.6 ± 5.6 and 87.5 ± 5.8 (weeks 3 and 4) % to 86. 1 ± 6.8 % (week 5). When sleep analysis was performed only between pre-LE and LE week 5, there were no significant differences (*p* > 0.36; Supplementary Table 5) for any of the parameters.

**Table 3:** Sleep variables derived from rest-activity recordings.

	**Pre – LE week**	**LE weeks**
Bedtime (clock time)	23.59 (1.19)	23.67 (1.31)
Sleep onset (clock time)	23.79 (1.18)	23.89 (1.32)
Wake time (clock time)	7.24 (0.99)	7.31 (1.05)
Get-up time (clock time)	7.33 (0.99)	7.39 (1.05)
Time in bed (h)	7.72 (0.74)	7.71 (0.77)
Sleep duration (h)	7.44 (0.73)	7.41 (0.77)
Wake duration (h)	0.68 (0.39)	0.69 (0.34)
Sleep efficiency (%) ∧	87.43 (6.42)	87.14 (5.77)
Sleep latency (h)	0.21 (0.16)	0.22 (0.16)

*All values are indicated as means and SD (in brackets) for the pre – light exposure (LE) week and LE weeks (i.e., average of weeks 2–5 when patients were exposed to bright light); n = 20. Bedtimes, sleep onset, wake and get-up times are indicated as clock time (h.mm), and time in bed as well as sleep duration and wakefulness during sleep episodes in hours (h.mm). Sleep efficiency (SE) derived from the ratio of sleep duration / time in bed is expressed as percentage (%). Sleep latency indicates the time range between bedtime and consolidated sleep (indirectly assessed via the inbuilt algorithm of the sleep analysis software). There were no statistically significant differences between pre-LE and LE weeks. ∧ = p < 0.05, indicating higher SE during weeks 3 and 4 than 5 (main effect of week; for mean values in weeks 3–5 separately see also text); n = 20*.

### Subjective Sleep Quality and Well-Being

**Figure 2:**
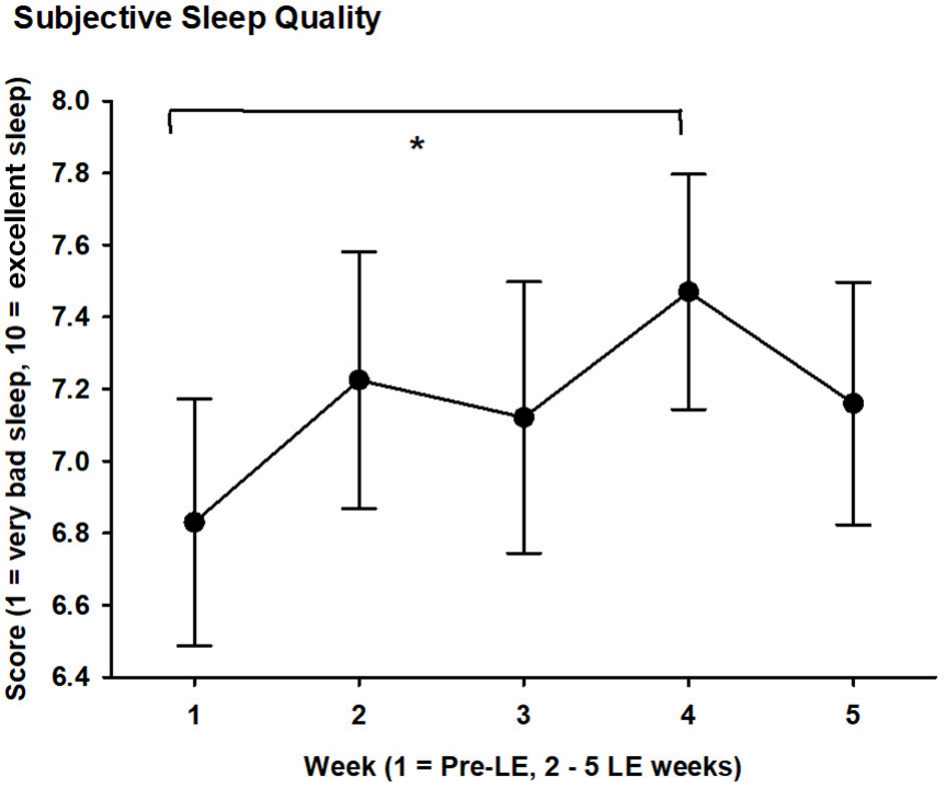
Subjective sleep quality score (1 = very bad sleep, 10 = excellent sleep) averaged per week during 5 weeks for 20 subjects with glaucoma. Mean values (black filled circles) and SEM are shown; LE = light exposure; week 1 = pre-LE week; weeks 2–5 = LE weeks. * = significant differences between weeks 1 and 4 (see text for mean value during week 4; *p* < 0.05).

Subjective sleep quality became significantly better for week 4 of the LE weeks (7.5 ± 1.5 mean ± SD) when compared to the pre-LE week (6.8 ± 1.5; *F*_4, 63_ = 2.7, *p* = 0.04; main effect of WEEK). Table 4a shows the values for the pre-LE week and the average of all LE weeks. For the time course of subjective sleep quality, see Figure 2. The Supplementary Figure 2 shows individual increases of subjective sleep quality in 15 of 20 participants during the LE weeks (subjective sleep quality data for week 3 was missing from one participant).

**Table 4a:** Subjective assessments for sleep quality [score 1 (very bad) – 10 (excellent)], and results from visual analog scales for relaxation (0 = extremely relaxed, 100 = extremely tense), physical comfort (0 = physically comfortable, 100 = physically not at all comfortable), alertness (0 = extremely alert, 100 = extremely sleepy) and mood (0 = bad mood and 100 = very good mood) on visual analog scales (0–100 mm).

	**Pre – LE week**	**LE weeks**
Subjective sleep quality^*^	6.83 (1.53)	7.25 (1.53)
Relaxation	37.6 (19.0)	36.6 (22.1)
Physical comfort	32.2 (17.7)	34.2 (20.3)
Alertness	35.9 (23.2)	32.7 (19.2)
Mood^*^	71.3 (16.7)	66.5 (18.2)

*LE = light exposure; all values are indicated as means and SD (in brackets) for the pre – LE week and the light LE weeks (i.e., average of weeks 2–5 when patients were exposed to bright light); n = 20*;

* *= p < 0.05, main effect WEEK*.

For relaxation, physical comfort and alertness there was no significant difference between pre-LE and LE weeks values. Mood decreased in the first LE week compared to pre-LE, but was not significantly different from pre-LE for the other 3 weeks (main effect of WEEK, *F*_4, 62_ = 2.7, *p* = 0.04).

### Visual Comfort

In general, the enhanced daily bright light exposure was well-tolerated by all participants and there were no negative reports from the light exposure such as headache, discomfort glare or eye strain. None of the 5 items of the visual comfort changed between the pre-LE week and the LE weeks (main effect of WEEK; *F*_4, 61_ <1.5, *p* > 0.22). All visual comfort scores were on average in the first half, that is, better comfort (item 1, 2, 5) or neutral (items 3, 4; i.e., in the middle, around 50; Table 4b). Visual comfort (and well-being) data from one participant was missing for 2 weeks during light exposure.

**Table 4b:** Subjective daily assessments for visual comfort (five items) on visual analog scales (1 – 100 mm).

	**Pre – LE week**	**LE weeks**
I like the light in this room (yes – no)	31.4 (17.0)	30.0 (17.3)
The light is comfortable (yes – no)	30.7 (17.0)	30.1 (17.4)
This room is too bright/too dark	50.7 (13.3)	47.2 (11.5)
There is too much light to read/there is not enough light to read	51.8 (13.8)	48.2 (10.6)
The glare in this room is imperceptible/intolerable	35.4 (16.4)	34.1 (20.5)

*LE = light exposure*.

*All values are shown as mean values and SD (in brackets) for the pre-LE week and LE weeks (i.e., average of weeks 2–5 when patients were exposed to scheduled daily bright light); n = 20*.

### Correlations

**Figure 3:**
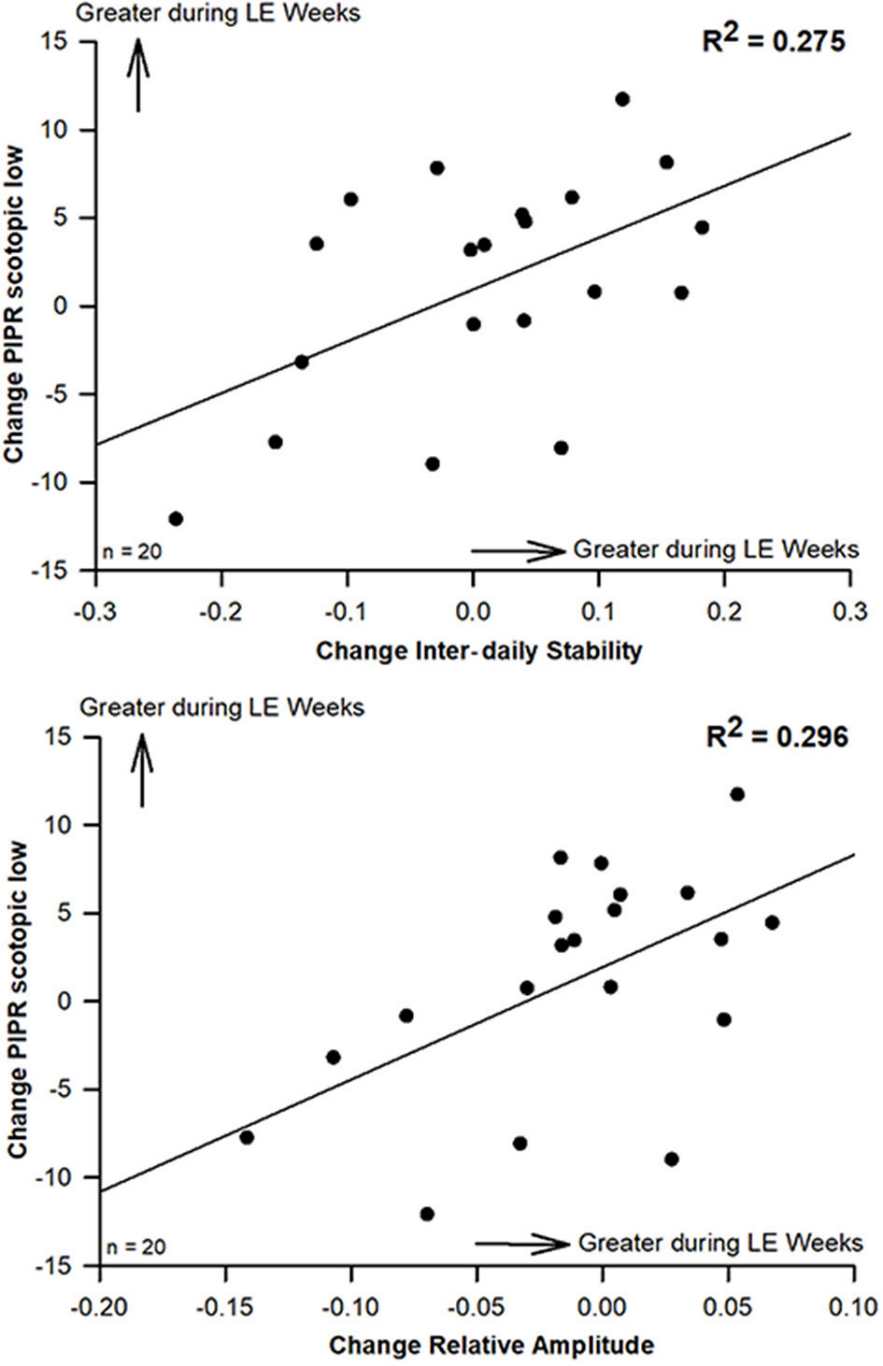
Scatterplots (and regression lines) for significant correlations between changes of the scotopic PIPR (to the lower luminance 1s light stimulus at 56 cd/m^2^) and inter-daily stability (IS; upper graph; *R*^2^ = 0.275) and with relative amplitude of rest-activity cycles (RA; lower graph; *R*^2^ = 0.296; Pearson's correlation; *n* = 20). Positive numbers indicate higher IS and RA as well as greater scotopic PIPR (to the 1s light stimulus at lower luminance; 56 cd/m^2^) during LE weeks (compared to pre-LE; *p* < 0.05; *n* = 20.

To correlate changes between the pre-LE and the LE week, values of the pre-LE week were subtracted from mean values of 4 LE weeks for the circadian and sleep related parameters as well as the subjective sleep quality and the VAS. Also, the difference between pre-LE and post-LE was used for all pupil parameters from two recordings, and the same was also done for the PSQI. There were significant correlations for increased PIPR (at lower luminance; 56 cd/m^2^) during LE weeks with greater inter-daily stability (IS; *R*^2^ = 0.275; *p* = 0.018; Figure 3, upper graph) and greater relative amplitude of 24-h rest-activity cycles (RA; *R*^2^ = 0.296; *p* = 0.013; Figure 3, lower graph) during LE-weeks. A statistical trend in the same direction was observed for the higher luminance PIPR (170 cd/m^2^) and relative amplitude (*p* = 0.07; *R*^2^= 0.17). An increase in subjective sleep quality during LE was correlated with a lower global PSQI score post LE (a lower PSQI is better), which suggests, that both assessments went in the same (positive) direction (*p* = 0.04; *R*^2^= 0.21; see Supplementary Figure 3, upper graph). An increase in subjective mood during the LE weeks was correlated with earlier wake time (*p* = 0.03; *R*^2^= 0.23; see Supplementary Figure 3, lower graph).

## Discussion

In a small group of patients with glaucoma of heterogeneous types and without severe visual loss, we found that increasing their daily light exposure during daytime by adding a table-based light box in their home had a favorable effect on subjective sleep quality and increased their melanopsin dependent pupil response (PIPR). This effect was observed after only 4 weeks. The scheduled bright light exposure, which was added to habitual light exposure for 30 min daily, did not alter or interfere with the daily activities of the participants. The additional light exposure did not alter timing or duration of habitual sleep nor did it change 24-h rest-activity cycles (both assessed from activity recordings). There was an increase in PIPR at the end of 4 weeks of light exposure and there was a significant correlation between the change in PIPR and the change in 24-h amplitude and inter-daily stability of rest-activity cycles during light exposure.

In line with our hypothesis, we found the melanopsin-mediated pupil response, the PIPR, was greater after 4 weeks of additional daytime light exposure. This change in PIPR suggests that in patients with glaucoma, the melanopsin activity in viable ipRGCs can adapt to different light levels if sustained over a certain period. Why might we think that additional daytime light exposure over 4 weeks is an adaptation response of the melanopsin system? In a previous study, we had demonstrated that the PIPR to a bright narrow-bandwidth short wavelength light modulates with long-term changes in light timing, such as seasonal changes of daylight (36, 47). In the current study, we did not change the light exposure timing, but enhanced brightness of light exposure in the morning over 4 weeks. We found a greater PIPR at the end of 4 weeks without any substantial change in timing of rest-activity, sleep duration or 24-h amplitude as assessed from rest-activity recordings. The significant correlation with higher relative amplitude and inter-daily stability (both derived from rest-activity cycles over 4 weeks) with higher PIPR after LE weeks suggests that there may be a common mode of action conveyed by melanopsin, even though this correlation is not taken as evidence for causality.

We cannot exclude the possibility that the increase in subjective sleep quality is a “placebo” effect or linked to other behavioral factors as it may be the decrease of mood between the pre-LE week and the first week with LE. The absence of change in the objective sleep (or circadian) parameters, assessed from the activity recordings, when compared to the pre-LE week might be taken as support for a placebo effect, especially since we did not ask participants to adhere to a certain sleep-wake schedule. We would argue that this seeming discrepancy in the subjective vs. objective sleep evaluation may be a methodologic one. Given the nature of this field study, we used rest-activity recordings as an indirect objective measure of sleep whereas the studies assessing sleep in a laboratory setting with standardized means, that is, polysomnography, have demonstrated that improved subjective sleep quality implicates objectively improved sleep. These studies found that sleep continuity and rapid eye movement (REM) sleep were correlated with subjective sleep quality (48) and that slow wave sleep (“deep sleep”) was the best predictor for subjective sleep quality (49).

Another minor but interesting result of our study was the correlation between changes toward earlier wake times during the LE weeks and better subjective mood when compared to pre-LE. This goes along with well-established evidence for light therapy outcomes for patients with mood disorders. Benedetti and colleagues for example showed, (50) that a combination of light therapy and sleep deprivation resulted in antidepressant effects which were correlated with circadian phase advances of rest-activity acrophases. Even though our patients with glaucoma were not depressed, and we did not determine circadian phase or found a difference in their sleep timing over 4 weeks, the correlation with an earlier wake time and better mood during the LE weeks (compared to pre-LE) points in a similar direction, but will certainly need further consideration to confirm improved non-visual functions in patients with ipRGC loss. We recognize that 10 of 20 patients had an artificial intraocular lens (non-blue-light blocking) which permits greater transmission of the light into the eye compared to the natural lens of the other 10 patients. However, a previous study had shown no correlation between lens transmission of blue light and pupil response (51). The current study assessed the change in pupil response before and after additional daytime light in a within subject-design, thus lens status is not likely to be a confounding variable to our results.

A potential concern regarding use of a light box might be the “blue light hazard.” Indeed there is a photochemical risk to the retinal tissues of the eye associated with ocular exposure to bright light sources such as the sun or welding arcs (52). The term “blue light hazard” defines the optical radiation risk for photochemical injury which peaks in the short-wavelength (“blue”) part of the optical radiation spectrum around 435 to 440 nm. The International Commission on Illumination (Commission Internationale de l' Eclairage, CIE) published a standard on this [as part of the CIE S 009:2002 “*Photobiological safety of lamps and lamp systems,”* (53)] and states in its position statement:…'*There is no evidence in humans of any adverse health effects from occasional exposure to optical radiation at the exposure limits*.'…(54). The lamps in our study emitted a broad spectrum white light and exposure followed the manufacturer guidelines. Clinical follow-up of patients within months of study participation did not demonstrate evidence of retinal toxicity.

This study is not a call for the use of light therapy for patients with glaucoma. Rather, the study results lend further support to the notion that patients in whom ipRGCs are damaged or dysfunctional due to glaucoma (or other neuroretinal disorders) may benefit from enhanced daytime light exposure which serves as a more effective zeitgeber. In turn, better distinction of their biological day and night may influence active synchronization of the internal circadian clocks with the external 24-h day (also called entrainment). While more daytime light may seem like obvious good advice, it is not always apparent and in practice. Most persons are indoors during many hours of the day (school, workplace, home) and office lighting is far less bright than natural daylight. Elderly persons tend to remain indoors for a variety of reasons: medical problems such as unsteady gait or poor vision, social issues such as fear of public transport, social isolation or confined living in an institution and personal comfort such as getting cold easily. This study has demonstrated that even a rather short duration of added light exposure (30 min) in a room is beneficial and supports the general advice to elderly persons to sit outdoors or go outside for 30 min each morning.

Thus, while glaucoma patients can never recover the vision lost from damaged retinal ganglion cells, they may be able to maintain a robust day–night cycle and concomitant good circadian entrainment which helps maintain high sleep quality. This will indirectly support good daytime performance and a sense of well-being. All these effects together might reduce vulnerability for other co-morbidities such as depression and disordered sleep. For this reason, further studies examining optimized light (intensity and timing) for glaucoma patients are needed.

## Data Availability Statement

The raw data supporting the conclusions of this article will be made available by the authors, without undue reservation.

## Ethics Statement

The studies involving human participants were reviewed and approved by Commission d'Ethique de Recherche sur l'être humain de Canton de Vaud, Switzerland no. 2018-01749. The patients/participants provided their written informed consent to participate in this study.

## Author Contributions

AK and MM designed the experiment. MU and ME performed the study. AK, MU, and MM analyzed the data. AK and MM wrote the manuscript. All authors reviewed and approved the final version of the manuscript.

## Conflict of Interest

The authors declare that the research was conducted in the absence of any commercial or financial relationships that could be construed as a potential conflict of interest.
