# Supplementary material for: Can Extra Daytime Light Exposure Improve Well-Being and Sleep? A Pilot Study of Patients With Glaucoma
Source: Front Neurol. 2021 Jan 15;11:584479. doi: 10.3389/fneur.2020.584479 (PMC7843442; doi:10.3389/fneur.2020.584479)
Supplement: Supplementary file 1 [file Data_Sheet_1.pdf]

**Supplementary Figure 1**

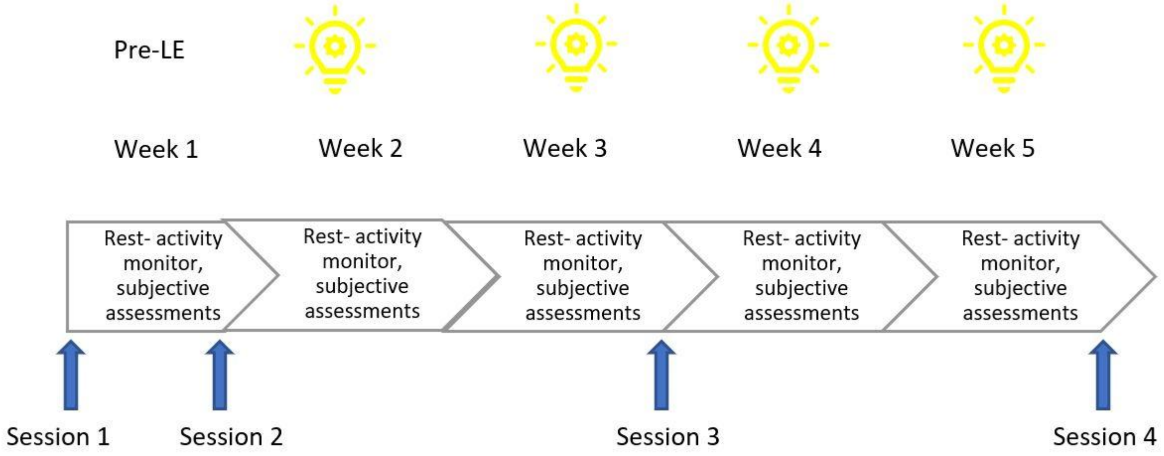


**Supplementary Figure 1:** Schematic study overview with the 4 sessions, where participants came to the laboratory. The pre-light exposure (LE) week and 4 LE weeks were spent at home, under normal daily routines while continuously recording rest-activity cycles and daily completion of self-assessments for sleep quality, mood, alertness, wellbeing and visual comfort. Pupillometry was performed during session 2 and 4. The yellow bulbs indicate the LE weeks with scheduled daily bright LE for 30 min.

**Supplementary Figure 2**

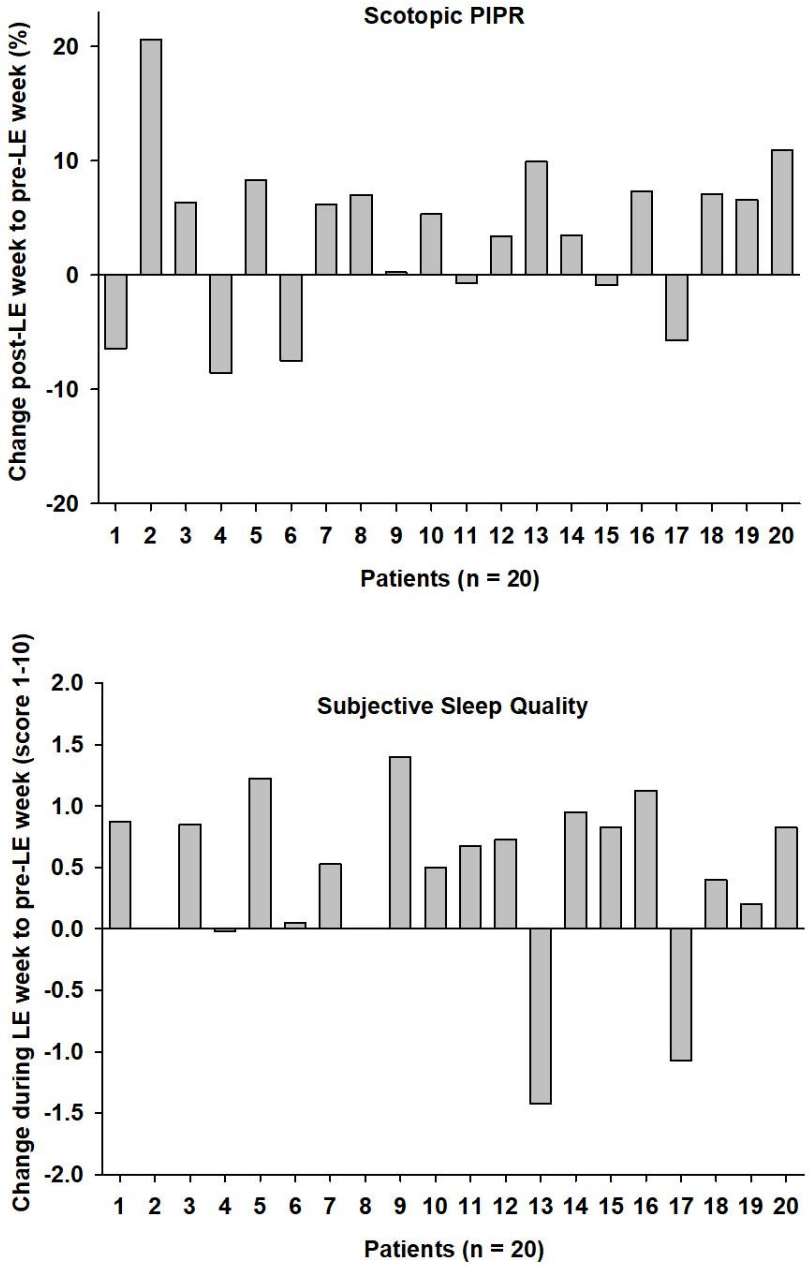


**Supplementary Figure 2:** The upper graph shows individual differences of the scotopic PIPR at higher luminance (170 cd/m$^2$; upper graph) between the pre-LE and post-LE (LE= light exposure). The lower graph shows individual differences in subjective sleep quality between the pre-LE (average of week 1) and LE weeks (average between weeks 2-5). The subjective sleep quality scores derived from the daily completed visual analogue scales for sleep quality ranging between 1 = very bad sleep and 10 = excellent sleep. The differences on both graphs are shown for each participant (1-20). Positive values indicate greater scotopic PIPR and greater subjective sleep quality (n = 20).

**Supplementary Figure 3**

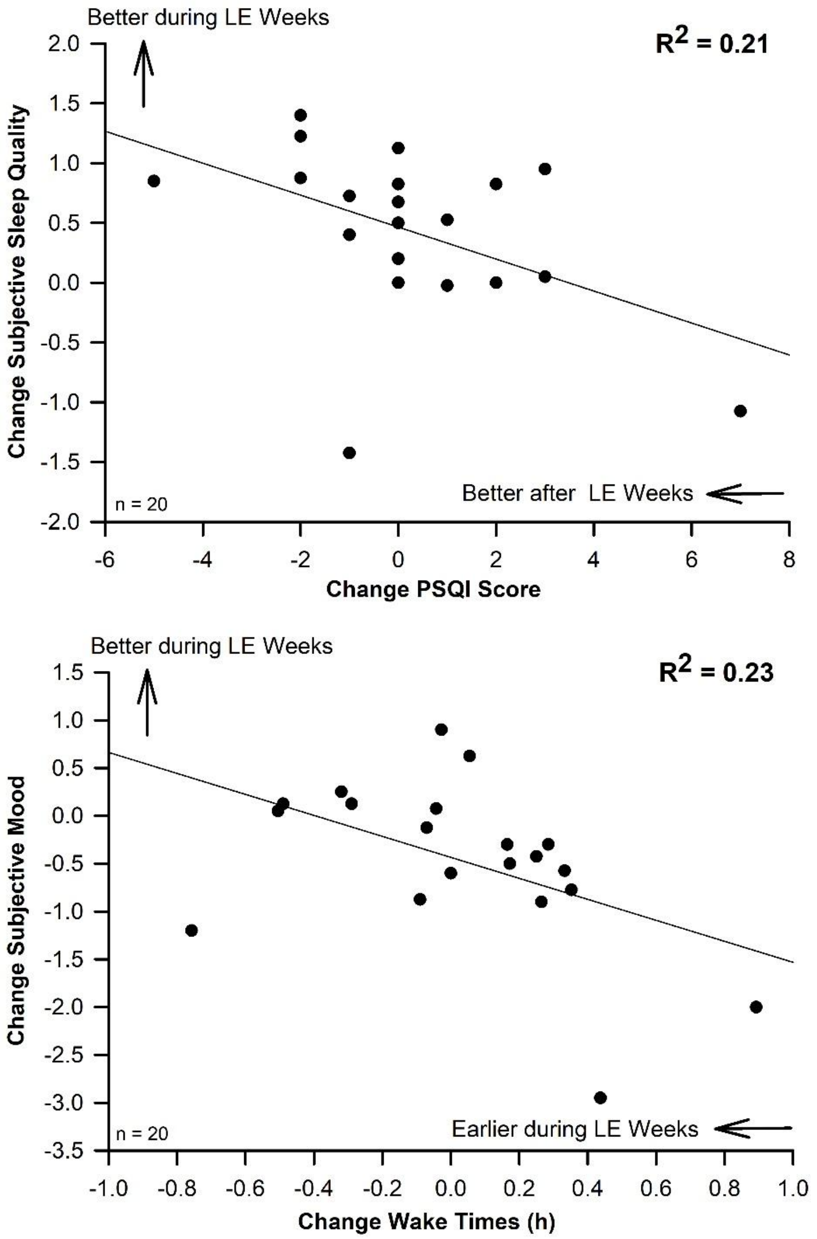


**Supplementary Figure 3**

Upper graph: Scatterplots (and regression lines) with correlation analysis for changes of the PISQI and subjective sleep quality ($p = 0.04$; $R^2 = 0.21$) during LE weeks compared to pre-LE. Lower graph: correlation between changes of habitual wake time and subjective mood (from VAS) during LE weeks compared to pre-LE ($p = 0.03$; $R^2 = 0.23$; Pearson's correlation; n = 20). The direction of effects is indicated with arrows in the respective corner of the upper and lower graph; n = 20.

**Supplementary Table 1**

| | **Mean (SD)** |
|---|---|
| Age (years) | 67.55 (7.45) |
| Sex (Female/Male) | 9/11 |
| VA RE | 0.80 (0.19) |
| VA LE | 0.89 (0.22) |
| Mean VA (both eyes) | 0.85 (0.17) |
| Octopus MD RE (dB) | 5.58 (4.77) |
| Octopus MD LE (dB) | 5.24 (3.85) |
| Mean Octopus MD (both eyes) | 5.41 (3.17) |
| OCT RNFL RE (A; B; N) | 17A; 2B # |
| OCT RNLF LE (A; B; N) | 18A; 1N # |

Demographics and eye examination results, shown as mean values ± Standard Derivations (SD; in brackets; n = 20). RE = right eye; LE = left eye; VA = visual acuity; MD = mean deviation; OCT = optical coherence tomography; RNFL = thickness of retinal nerve fiber layer; A = OCT RNFL abnormal; B = OCT RNFL borderline; N = OCT RNFL normal; #: n=19 (one patient’s data missing).

**Supplementary Table 2**

| | **Mean (SD; range)** |
|---|---|
| MCTQ (MSF-Sc) | 3.0 (1.1; 1.25 - 5.29) |
| ESS | 5.8 (4.4; 0 - 19) |
| HO | 64.0 (9.0; 45 - 82) |
| SPAQ | 5.2 (5.5; 0 - 16) |
| BDI | 3.1 (3.0; 0 - 10) |
| PSQI[a, b] | 5.5 (3.4; 1 - 12) / 5.7 (4.1;1 - 14) |

Results from screening questionnaires are shown as mean (± SD; range, in brackets) values for: the Munich Chronotype Questionnaire (MCTQ; MSF-Sc = midsleep on free days, sleep duration corrected); Epworth sleepiness scale (ESS); Horne Ostberg (HO); Seasonal Pattern Assessment Questionnaire SPAQ); Becks Depression Inventory (BDI); Pittsburgh Sleep Quality Index (PSQI); [a] = mean PSQI post-light light exposure; [b] = not significant ($p > 0.67$); $n = 20$.

**Supplementary Table 3**

| irradiance, mW/m$^2$ | illuminance, lx | log photon irradiance, log10/s/cm$^2$ |
|---|---|---|
| 10357.53 | 3547.97 | 15.462 |

| | S-cone-opic | M-cone-opic | L-cone-opic | Rhodopic | Melanopic |
|---|---|---|---|---|---|
| α-opic irradiance, mW/m$^2$ | 1943.86 | 4591.69 | 5673.87 | 3410.49 | 2709.43 |
| α-opic equivalent daylight (D65) illuminance (α-opic EDI), lx | 2378.42 | 3154.01 | 3483.24 | 2352.54 | 2042.98 |
| α-opic log photon irradiance, log/s/cm$^2$ | 14.641 | 15.097 | 15.209 | 14.938 | 14.824 |

Measurements of the light source (EnergyUp I/ HF3419™ Philips, The Netherlands) taken in 50 cm distance at the vertical eye level of a user with a spectroradiometer (Specbos 1201, Jeti, Jena, Germany). The alpha-opic (α-opic) units are based on the standard of the Commission International de l’ Éclairage (CIE S 026) and calculated by using the Toolbox beta version E1.051 – 20190319.

**Supplementary Table 4**

| Baseline Pupil Sizes | Pre-LE Right Eyes | | Pre-LE Left Eyes | | Post-LE Right Eyes | | Post-LE Left Eyes | |
|---|---|---|---|---|---|---|---|---|
| Photopic (low) | 3.50 | (0.73) | 3.47 | (0.66) | 3.59 | (0.71) | 3.49 | (0.66) |
| Photopic (high) | 3.41 | (0.73) | 3.52 | (0.61) | 3.51 | (0.72) | 3.58 | (0.63) |
| Scotopic (low) | 5.07 | (0.76) | 4.97 | (0.82) | **5.14*** | **(0.77)** | **4.86** | **(0.86)** |
| Scotopic (high) | 4.61 | (0.73) | 4.48 | (0.64) | **4.64*** | **(0.64)** | **4.39** | **(0.72)** |

Baseline (BL) pupil sizes [mm; mean (SD, in brackets)] obtained before photopic and scotopic light pupil test stimuli at two different luminance levels: 56 cd/$m^2$ (indicated as low) and 170 cd/$m^2$ (indicated as high), for pre-LE week (LE = light exposure; i.e. before the 4 weeks with daily scheduled bright light exposure) and post-LE (i.e. after 4 weeks of daily scheduled bright light exposure). * = $p < 0.05$; with smaller baseline pupil sizes for left than right eyes (significant results are shown in bold); n = 20.

**Supplementary Table 5**

| | PRE-LE week | LE week 5 |
|---|---|---|
| | | |
| **Bedtime** | 23.59 (1.19) | 23.61 (1.30) |
| **Sleep Onset** | 23.79 (0.26) | 23.88 (0.29) |
| **Wake Time** | 7.24 (0.22) | 7.28 (025) |
| **Get up Time** | 7.33 (0.99) | 7.35 (1.14) |
| **Time in Bed** | 7.72 (0.74) | 7.73 (0.94) |
| **Sleep Duration** | 7.44 (0.73) | 7.38 (0.91) |
| **Wake Duration** | 0.68 (0.39) | 0.72 (0.32) |
| **Sleep Efficiency (%)** | 87.43 (6.42) | 86.13 (6.09) |
| **Sleep Latency** | 0.21 (0.16) | 0.27 (0.23) |

Sleep variables derived from rest-activity recordings (same variables as Table 3 of the main text); all times are indicated in decimal clock time or hours; LE = light exposure; all values are indicated as means and SD (in brackets) and shown for the PRE-LE week and LE week 5 (i.e. the last week of daily scheduled bright light exposure), n = 20 (there were no statistically significant differences between both weeks; $p > 0.36$).
